# WNT-inhibitory factor 1-mediated glycolysis protects photoreceptor cells in diabetic retinopathy

**DOI:** 10.1186/s12967-024-05046-5

**Published:** 2024-03-06

**Authors:** Bolin Chen, Jing Zou, Lihui Xie, Yinjun Cai, Bowen Li, Wei Tan, Jinhaohao Huang, Fangling Li, Huizhuo Xu

**Affiliations:** 1 Eye Center of Xiangya Hospital, Hunan Key Laboratory of Ophthalmology, Central South University, No 87, Xiangya Road, Kaifu District, Changsha, 410008 Hunan China; 2 National Clinical Research Center for Geriatric Disorders, Xiangya Hospital, Central South University, Changsha, 410008 Hunan China; 3 Department of Ophthalmology, Xiangtan Central Hospital, Xiangtan, 411199 Hunan China

**Keywords:** Diabetic retinopathy, Photoreceptor cell, Retinal neuronal degeneration, Wnt-inhibitory factor 1, Glycolysis

## Abstract

**Background:**

In diabetic retinopathy (DR), hypoxia-inducible factor (HIF-1α) induces oxidative stress by upregulating glycolysis. This process leads to neurodegeneration, particularly photoreceptor cell damage, which further contributes to retinal microvascular deterioration. Further, the regulation of Wnt-inhibitory factor 1 (WIF1), a secreted Wnt signaling antagonist, has not been fully characterized in neurodegenerative eye diseases. We aimed to explore the impact of WIF1 on photoreceptor function within the context of DR.

**Method:**

Twelve-week-old C57BL/KsJ-db/db mice were intravitreally injected with *WIF1* overexpression lentivirus. After 4 weeks, optical coherence tomography (OCT), transmission electron microscopy (TEM), H&E staining, and electroretinography (ERG) were used to assess the retinal tissue and function. The potential mechanism of action of WIF1 in photoreceptor cells was explored using single-cell RNA sequencing. Under high-glucose conditions, 661 W cells were used as an in vitro DR model. WIF1-mediated signaling pathway components were assessed using quantitative real-time PCR, immunostaining, and western blotting.

**Result:**

Typical diabetic manifestations were observed in db/db mice. Notably, the expression of WIF1 was decreased at the mRNA and protein levels. These pathological manifestations and visual function improved after *WIF1* overexpression in db/db mice. TEM demonstrated that WIF1 restored damaged mitochondria, the Golgi apparatus, and photoreceptor outer segments. Moreover, ERG indicated the recovery of a-wave potential amplitude. Single-cell RNA sequencing and in vitro experiments suggested that *WIF1* overexpression prevented the expression of glycolytic enzymes and lactate production by inhibiting the canonical Wnt signaling pathway, HIF-1α, and Glut1, thereby reducing retinal and cellular reactive oxygen species levels and maintaining 661 W cell viability.

**Conclusions:**

WIF1 exerts an inhibitory effect on the Wnt/β-catenin-HIF-1α-Glut1 glycolytic pathway, thereby alleviating oxidative stress levels and mitigating pathological structural characteristics in retinal photoreceptor cells. This mechanism helps preserve the function of photoreceptor cells in DR and indicates that WIF1 holds promise as a potential therapeutic candidate for DR and other neurodegenerative ocular disorders.

**Graphical Abstract:**

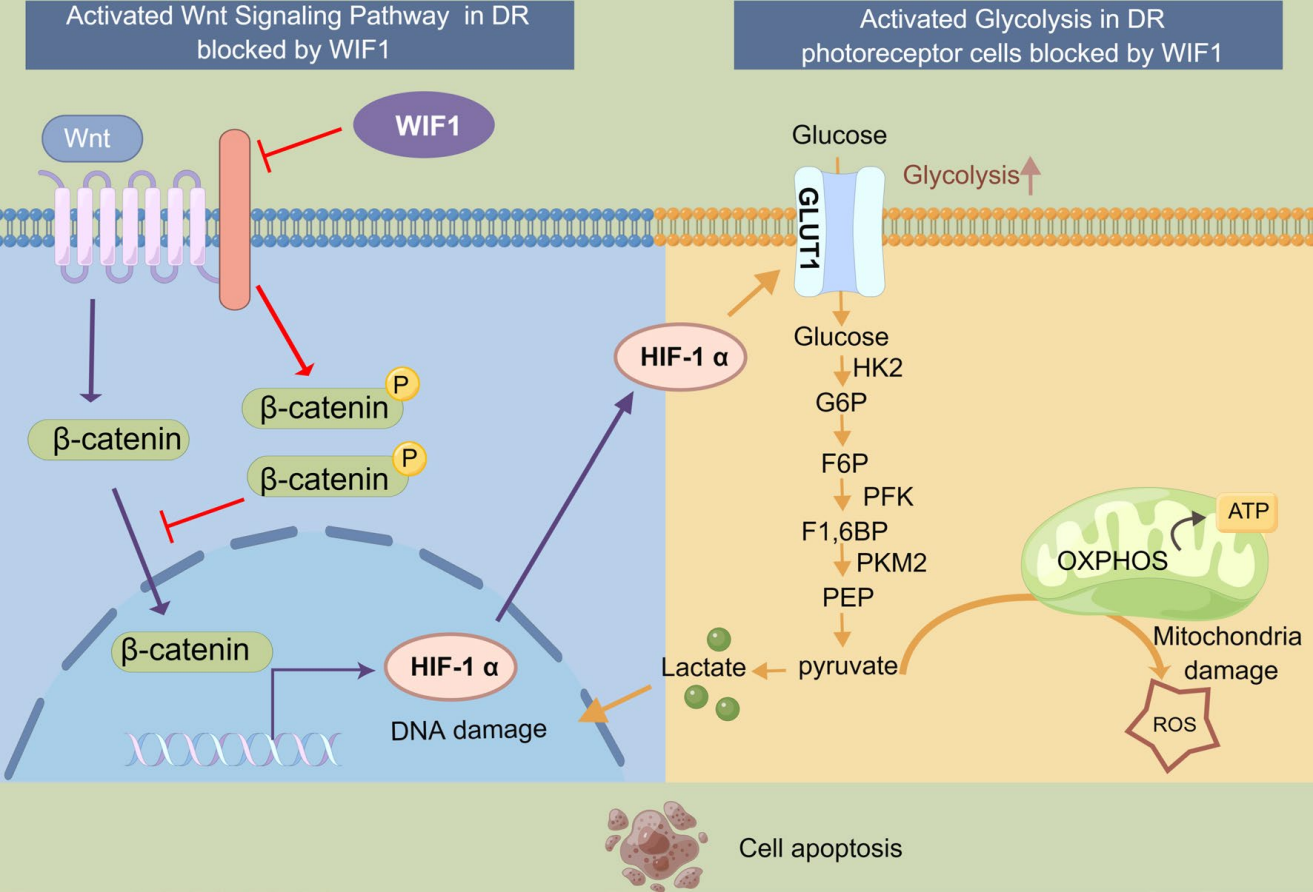

**Supplementary Information:**

The online version contains supplementary material available at 10.1186/s12967-024-05046-5.

## Highlights

- WIF1 inhibits HIF-1α-Glut1 glycolytic pathway in photoreceptor cells
- Improves oxidative stress and ameliorates pathological structural characteristics.
- Helps preserve the function of photoreceptor cells in diabetic retinopathy
- WIF1 is an excellent candidate for targeted therapy against diabetic retinopathy.

**Supplementary Information:**

The online version contains supplementary material available at 10.1186/s12967-024-05046-5.

## Introduction

Diabetic retinopathy (DR) is the most prevalent and specific complication associated with diabetes mellitus (DM), causing significant damage to the vision of working adults. By 2020, approximately 100 million people had been diagnosed with DR globally, and this number is anticipated to rise to 160 million by 2045 [1, 2]. The primary characteristics of DR include microvascular leakage and retinal obstruction caused by persistently elevated blood glucose levels, coupled with the progressive degeneration of neuronal cells within the retina [3, 4]. Retinal neuronal degeneration occurs in the initial stages of DR; hyperglycemia induces changes in the metabolic retinal environment, which results in oxidative stress and inflammation, causing damage to neurons and glial cells and activating neurodegenerative processes. Oxidation and inflammation also disrupt the production and release of neurotrophic factors, further disrupting neurodegenerative processes. If left untreated, neurodegenerative processes and neovascularization occur simultaneously, resulting in visual dysfunction, ultimately leading to vision loss [5–7]. This indicates that early retinal neuron degeneration is an important factor in the progression of DR. However, presently, the clinical treatment for DR mainly targets reducing retinal neovascularization. Hence, it is both urgent and necessary to reveal the complex pathogenesis of DR neuron degeneration, providing new insights for the early intervention and clinical treatment of DR and designing novel therapies.

The hypoxia-inducible factor 1α (HIF-1α) is a crucial pathogenic factor that contributes to the development of DR. The expression of HIF-1α significantly increases in hypoxic environments, leading to the upregulated expression of numerous downstream target genes, including *vascular endothelial growth factor (VEGF)* and those coding glycolytic enzymes and membrane transporters [8]. This upregulation, in turn, increases glycolytic flux and enhances glycolysis, ultimately fostering oxidative stress. Oxidative stress leads to excessive reactive oxygen species (ROS) production, and long-term ROS production can induce metabolic abnormalities and damage to retinal cells, thereby exacerbating neurodegeneration in DR [9].

The Wnt signaling pathway is of substantial importance in the pathogenesis of eye disorders. Wnt-inhibitory factor 1 (WIF1) functions as an antagonist of the Wnt signaling pathway. It interacts directly with Wnt ligands, initiating the phosphorylation and subsequent degradation of the critical molecule β-catenin within the pathway, ultimately inhibiting the Wnt/β-catenin signaling pathway [10]. Hunter et al. reported that WIF1 is expressed during and after photoreceptor morphogenesis in mice, regulating mammalian photoreceptor cell development [11]. Park et al. demonstrated a correlation between the degree of structural impairment to the retinal photoreceptor cells and the level of WIF1 protein secreted by the aqueous humor in individuals with neovascular age-related macular degeneration (nAMD) [12]. Additionally, we previously demonstrated a progressive downregulation of *WIF1* in an oxygen-induced retinopathy (OIR) animal model. Notably, overexpression of *WIF1* in the vitreous OIR mice strongly attenuated photoreceptor cell injury [13].

Based on the existing literature, we hypothesized that WIF1 assumes a pivotal role in the regulation of neurodegenerative diseases within the context of DR, with particular emphasis on the impairment of photoreceptor cells and the function of neurons in the DR, which is affected by WIF1. Therefore, in the present study, we evaluated whether WIF1 could improve the oxidative stress levels and pathological structural characteristics of retinal photoreceptor cells in db/db mice, thereby alleviating DR. We also performed in vitro cellular experiments to explore the potential regulatory mechanism of action of WIF1. This study provides new insights and identifies potential targets for the treatment of DR.

## Material and methods

### Animal information

Male (12 week old) C57BL/KsJ-db/db and C57BL/KsJ-db/m mice were obtained from Jiangsu Jicui Yaokang Biotechnology Co., Ltd. (Jiangsu, China). The animals were raised in the Center of Laboratory Animals at Central South University (Changsha, China) and maintained under controlled temperature and illumination.

### Cell culture and treatment

Aolu Biotechnology (Shanghai, China) provided the mouse photoreceptor-derived (661 W) cell line. Cells were cultured using high-glucose Dulbecco’s modified Eagle’s medium (DMEM; NCM Biotech, Newport, RI, USA) with 10% fetal bovine serum (FBS; Gibco, Thermo Fisher Scientific, Waltham, MA, USA) and then incubated at 37 °C with 5% CO_2_. In vitro cell models of DR were created by supplementing the cell culture medium with 50 mM glucose (HG group) for 24 h. Another group of 661 W cells treated with 25 mM glucose served as the normal group. For the inhibition or activation of HIF-1α, 10 µM LW6 (S8441; Selleck; Houston, TX, USA) and 10 µM CoCl_2_ (S9490, Selleck) were added to cells. After 24 h, subsequent experiments were conducted.

### Intravitreal lentivirus administration

The lentiviruses were obtained from OBIO Technology (Shanghai, China). At the age of 12 weeks, anesthesia was administered to the db/db mice, followed by pupil dilation utilizing a 1% atropine solution. Next, utilizing a 33-gauge needle (Hamilton, Reno, NV, USA) under the guidance of a surgical microscope, lentiviruses were intravitreally administered 1 mm posterior to the limbus. Employing a randomized allocation method, the db/db mice were categorically distributed into distinct groups: control, LV-vector injected into the eyes; WIF1, LV-WIF1 carrying 3xFlag tag (DYKDHDGDYKDHDIDYKDDDDK) injected into the eyes; and db/db: no intervention was applied. After 4 weeks, ocular samples were procured to facilitate various analytical assessments.

### Isolation of retinal cells for single-cell RNA sequencing (scRNA-seq)

Four weeks after Lentivirus administration, three retinas were collected from the db/m, db/db, and WIF1 groups, combined as mixed samples, and then rinsed with precooled PBS. The tissue was thoroughly minced using surgical scissors and placed in a freshly prepared enzymatic solution. The enzymatic digestion occurred in a constant temperature incubator at 37 °C for 60 min. Cell concentration and viability were measured using a Luna cell counter (Logo Biosystems, Gyeonggi-do, South Korea). Cell viability exceeded 90% in all samples.

### scRNA-seq data preprocessing and quality control

The scRNA-seq libraries were generated using the Chromium Next GEM Single Cell 3ʹ Reagent Kit (1000268; 10 × Genomics, Pleasanton, CA, USA, version 3.1. Sequencing was performed on the Nova 6000 PE150 System (Illumina Inc., San Diego, CA, USA). The CellRanger software pipeline (version 7.0.1) was used to process the sequencing data. The Seurat package (version 4.3.0) in R (version 4.2.2) was used to integrate and cluster the data. Cells of substandard quality were eliminated, encompassing those featuring fewer than 200 genes and a mitochondrial gene ratio surpassing 40%.

### Dimensionality reduction and clustering analysis

The counts of individual cells were log-normalized using the ‘NormalizeData’. Dimensionality reduction was performed via the ‘RunPCA’. Cell visualization was accomplished through a two-dimensional UMAP algorithm implemented in the ‘RunUMAP’. Significant clusters were determined using ‘FindNeighbors’ and ‘FindClusters’. Marker genes for each cluster were identified using ‘FindAllMarkers’.

### Gene ontology (GO) and Kyoto encyclopedia of genes and genomes (KEGG) analyses

The online Metascape platform (www.metascape.org) was employed to conduct GO and KEGG pathway analyses using the input of differentially expressed genes (DEGs). Within the leading 50 enriched GO terms spanning various cell types, 10 GO terms or pathways linked to diseases were pinpointed using the ggplot2 package (version 3.4.1) within the R programming environment.

### DEG analysis

DEGs were detected utilizing the Find Marker function within the Seurat software. Significantly differential expression was defined with a P value < 0.05 and |log2fold change|> 0.25 as the established thresholds.

### Western blot analysis

Retinal tissues from mice along with 661 W cells were procured and subsequently subjected to treatment with a lysis buffer. Protein samples were separated using SDS-PAGE and transferred onto polyvinylidene difluoride (PVDF) membranes (#IPVH00010; Millipore, Burlington, MA, USA). The membranes were blocked with 5% nonfat milk for 2 h and incubated with primary antibodies. The samples were then incubated with horseradish peroxidase-conjugated rabbit anti-mouse IgG secondary antibody (511203; Zenbio, China) for 1 h at 22 ℃. After washing the membrane, the protein band was detected using the ChemiDoc system (BIO-RAD MP500; Bio-Rad Laboratories, Hercules, CA, USA). Protein bands on the membranes were observed using ImageJ software. The catalog numbers of all primary antibodies used are listed in Additional file 2: Table S2.

### Quantitative real-time PCR

Total RNA was isolated from retinal tissues and 661 W cells using a Yishan kit (Yishan, Shanghai, China). Quantitative real-time PCR (qRT-PCR) assays were performed as previously described [14]. The primers are listed in Additional file 3: Table S1.

### Electroretinography (ERG)

Mice were maintained in the dark for 12 h before being anesthetized using 1% sodium pentobarbital (50 mg/kg). ERG was recorded using an electrophysiological detector (RETI Port/Scan 21; Roland Co., Germany). ERG data was recorded using Full-field stimulation. Scotopic 1.0 cd s/m^2^, 3.0 cd s/m^2^, and 10.0 cd s/m^2^ were recorded in our study. The duration of each white flash stimulus was 5 ms. The implicit times of the a-waves were the measurements of the interval between the onset of the visual stimulus and the culmination of the a-wave. The amplitude was ascertained by calculating from the pre-stimulus baseline to the nadir of the waveform.

### Optical coherence tomography (OCT)

After satisfactory anesthesia, the pupils were dilated using 1% atropine. A viscoelastic agent was applied to the eyes of the mice to prevent dehydration. Using Phoenix Image-guided OCT (Phoenix Technology Group, Pleasanton, CA, USA), the mice were placed on a bracket. The lens was aimed at the eyeball and slowly approached; the angle was carefully adjusted to place the eyeball at the center of the lens. After adjusting the gamma value, OCT images of the optic nerve and surrounding area were obtained. Retinal layer segmentation was performed with InSight software (version v2.1.7237). The automated analysis results were measured by Fangling Li and Lihui Xie, who were blinded to the analysis. The final value was determined as the average of their measurements.

### Hematoxylin and eosin (H&E) staining

Eyeballs were fixed in 4% paraformaldehyde (PFA) and underwent serial sectioning (5 μm in thickness) using a sagittal approach that traversed through the optic nerve head. These sections were then subjected to H&E staining. Retinal morphology images were captured using a light microscope. The measurement of retinal thickness occurred at intervals of 400 μm from the optic nerve head, facilitated by Case Viewer 2.4 software. Cell nuclei in the outer nuclear layer were counted using ImageJ software.

### Immunofluorescence staining and fluorescent microscopy

Mouse eyeballs were fixed in 4% PFA at 4 °C overnight. To prepare frozen sections, the eyeballs were immersed in sucrose and embedded in an OCT compound. Immunofluorescence staining was performed using primary antibodies. Sections were incubated with Alexa Flour 574 goat anti-mouse IgG (H + L) (A11005; Invitrogen, Thermo Fisher Scientific) or Alexa Flour 488 goat anti-rabbit IgG (H + L) (A11008; Invitrogen) for 1 h without light at room temperature. Then sections were stained with 4′,6-diamidino-2-phenylindole (DAPI; Solarbio Biotechnology, Beijing, China) for 5 min. Images were captured using a fluorescence microscope (DM5000B; Leica, Weltzar, Germany). ImageJ image analysis software was used to quantify relative fluorescence intensities in the images.

### Lactate measurements

The levels of secreted lactate in 661 W cells and mouse retinas were measured using a Lactate Assay Kit (obtained from Nanjing Jiancheng Bioengineering Institute).

### Reactive oxygen species (ROS) analysis

The ROS Colorimetric Assay Kit (E-BC-K138-F; Elabscience Biotechnology, Wuhan, China) was used according to the manufacturer’s protocol to conduct the ROS assay. ROS flow fluorescence results were analyzed using FlowJo software version (v10.6.2).

### Statistical analysis

Statistical analyses involving two-group comparisons were performed using Student’s *t*-test and those involving multi-group comparisons were performed using one-way analysis of variance (ANOVA) in GraphPad Prism (version 9.2; GraphPad Software).

## Results

### WIF1 expression is decreased in db/db mice

**Fig. 1:**
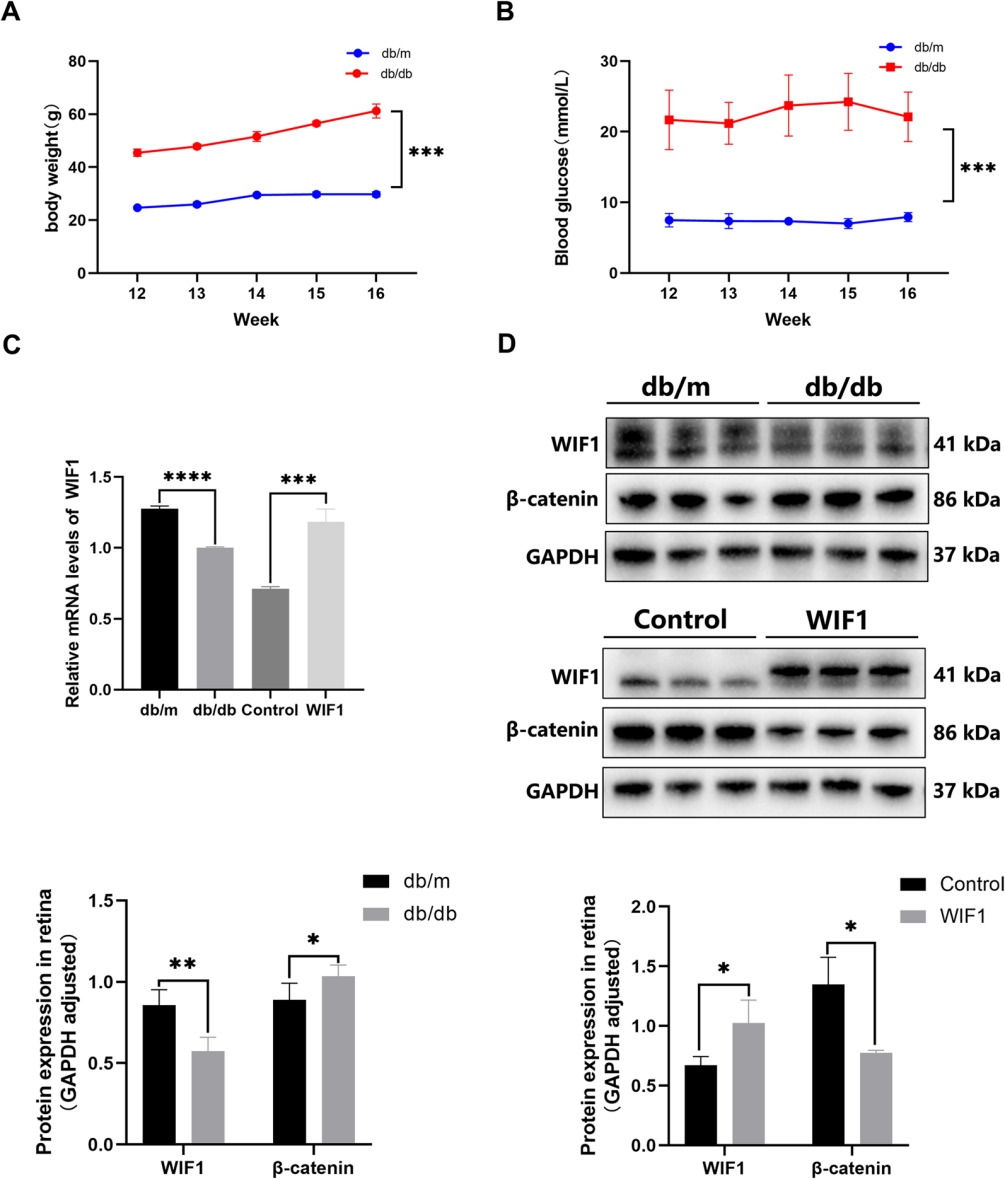
Expression level of WIF1 in db/db mice. **A** The body weight of db/m and db/db mice aged 12 to 16 weeks. **B** Blood glucose level of db/m and db/db mice aged 12 to 16 weeks. **C** qRT-PCR analysis of the mRNA levels of *WIF1*. **D** Western blot and analysis of retinal WIF1 and β-catenin protein levels in db/m, db/db, control, and WIF1 groups. Control: intravitreal injection control lentivirus of db/db mice. WIF1: intravitreal injection 3xFlag tagged WIF1 lentivirus of db/db mice. The presented data depict the average and standard deviation from a minimum of three repetitions, with differences assessed using standard one-way ANOVA. *p < 0.05, **p < 0.01, ***p < 0.001

We utilized C57BL/KsJ-db/db mice as a DR mode. Compared to db/m mice, db/db mice exhibited considerably increased body weight and fasting blood glucose starting at 12 weeks (Fig. 1A, B). qRT-PCR analysis revealed a more than twofold increase in *WIF1* mRNA levels within the retinas of db/db mice treated with WIF1 than those with the retinas of 16-week control db/db mice (Fig. 1C). Western blot analysis demonstrated an approximately 30% reduction in WIF1 protein levels and an upregulation of β-catenin in the retinas of 12-week-old db/db mice compared to those in the retinas of db/m mice. However, after 4 weeks of intravitreal injection with a lentivirus overexpressing *WIF1*, the expression of WIF1 was significantly increased, whereas that of β-catenin decreased (Fig. 1D).

### WIF1 alleviates photoreceptor cell injury in db/db mice

**Fig. 2:**
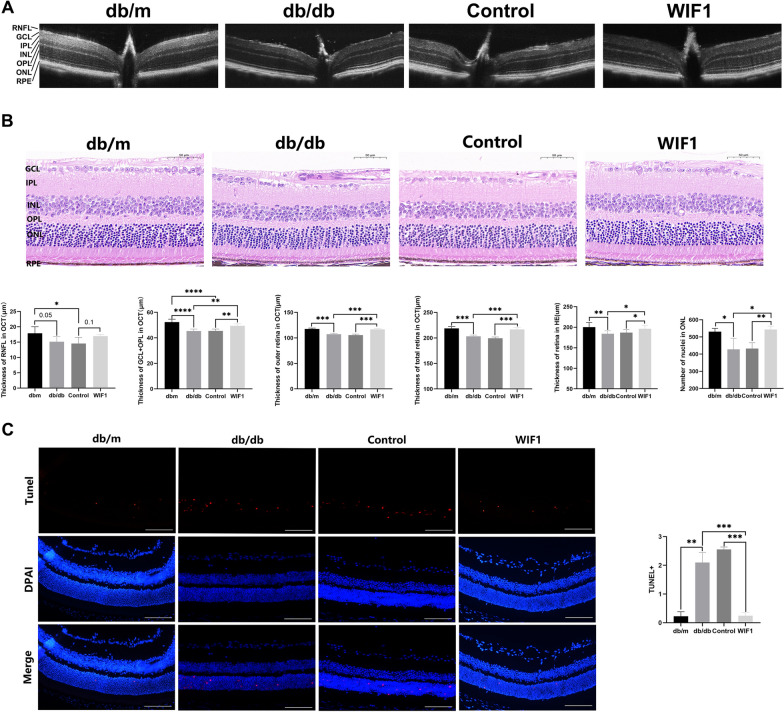
WIF1 overexpression has a positive protective effect on photoreceptor injury in db/db diabetic mice. **A** Representative image of retinal OCT in four groups of mice. Quantification of RNFL, GCL + OPL, total retinal thickness, and outer retinal thickness is shown within the circular region adjacent to the optic nerve head. n = 6 **B** Representative retinal H&E staining images for the four groups. Quantitative analysis of total retinal thickness within the circular area surrounding the optic nerve head and photoreceptor cell nuclei in the retinal ONL. Scale bar, 50 μm. n = 6 **C** Representative images depicting TUNEL (red) staining in retinal sections from the four groups of mice. The relative fluorescence ratio of TUNEL was subsequently analyzed. The presented data depict the average and standard deviation from a minimum of three repetitions, with differences assessed using standard one-way ANOVA. *p < 0.05, **p < 0.01, ***p < 0.001

To investigate the function of WIF1 in the DR context, we conducted OCT imaging, comparing retinal layers between age-matched (16 weeks) db/m and db/db mice. The measurements included both total thickness and the thickness of various segmented layers in each eye, taken at equidistant distances of 0.6–0.8 mm from the center of the optic nerve head [15]. We found that in db/db mice, the overall retinal thickness, extending from the internal limiting membrane (ILM) to the retinal pigment epithelium (RPE) layer was reduced, particularly in the outer layer of the retina, including the outer plexiform layer (OPL), outer nuclear layer (ONL), and inner segment-outer segment (IS/OS). The ganglion cell layer + inner plexiform layer (GCL + IPL) and retinal nerve fiber layer (RNFL) also exhibited a certain degree of reduction, and treatment with WIF1 reversed these effects (Fig. 2A). The OCT results suggest that WIF1 improved the retinal structure and reduced apoptosis.

H&E staining revealed a significant increase in retinal thickness, particularly evident in the increased number of photoreceptor cell nuclei within the ONL following one month of treatment with WIF1 in db/db mice (Fig. 2B). Terminal deoxynucleotidyl transferase (TdT) dUTP Nick-End Labeling (TUNEL) staining was used to evaluate apoptosis in retinal cells, and the fluorescence results indicated that WIF1 inhibited photoreceptor cell apoptosis in the ONL of db/db mice (Fig. 2C). These results preliminary suggest that WIF1 has a positive protective effect against photoreceptor cell injury in DR.

### scRNA-seq reveals that WIF1 alleviates photoreceptor cell injury via the HIF-1α and glycolytic pathways

**Fig. 3:**
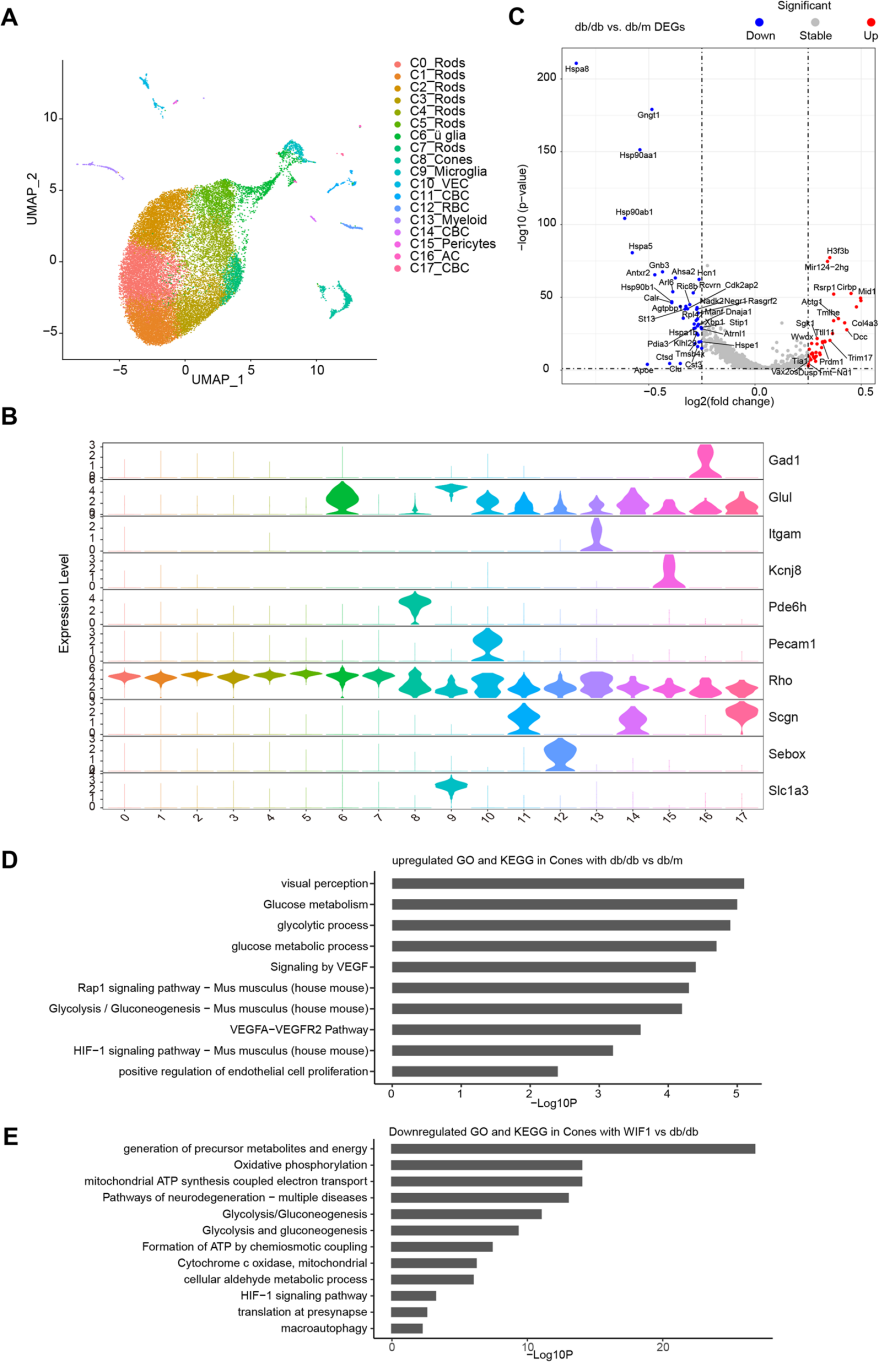
Single-cell RNA sequencing reveals the involvement of the Wnt, HIF-1, and glycolytic pathways in the mechanism of photoreceptor damage in db/db diabetic mice. **A** UMAP shows retinal cell grouping, which is divided into 10 subclasses according to gene marker, including rod cells (Rods, C0, C1, C2, C3, C4, C5, C7), Müller glia cells (ü glia, C6), cone cells (Cones, C8), microglia cells (Microglia, C9), retinal vascular endothelial cells, cone bipolar cells (CBC, C11, C14, C17), rod bipolar cells (RBC, C12), myeloid cells (myeloid, C13), pericytes (Pericytes, C15), amacrine cells (AC, C16); **B** Violin illustration of retinal cell grouping marker genes. Rods-Rho, Müller glial cells-Glul, cones-Pde6h, microglia-Slcla3, retinal vascular endothelial cells-Pecam1, cone bipolar cells-Scgn, rod bipolar cells-Sebox, myeloid cells-Itgam, pericytes-Kcnj8, amacrine cells-Gad1. **C** Volcano diagram of the upregulated differentially expressed genes (those associated with inflammation, apoptosis, autophagy, and ubiquitination: *Dusp*, *Cirbp*, *Actg1*, *Ttll11*, *Sgk1*, *and Mir124-2hg*) and downregulated ones (Hsp-family related genes: *Hsp90aa1* and *Hsp90ab1*). Genes with Log2 fold change > 0.25 and *P* < 0.05 were considered as differentially expressed genes. **D** Histogram showing the upregulation of signaling pathways in cone subsets in db/db mice compared to db/m mice (pre-Top30). VEGF, HIF-1, and glycolytic pathways were significantly upregulated. **E** Histogram showing the downregulation of signaling pathways (pre-Top30) in cone cells of WIF 1 mice compared to db/db mice. WIF1 can downregulate the glycolysis, HIF-1, and oxidative phosphorylation pathways, which are upregulated in db/db

To gain a deeper understanding of the regulatory mechanism of WIF1 in photoreceptor cells, we conducted scRNA-seq of db/db mouse retinas. We analyzed the distribution of four distinct cell lineages within the retina, encompassing six neuronal classes: cone bipolar cell (CBC), amacrine cell (AC), pericytes, rod, cone, and rod bipolar cell (RBC). We also analyzed two glial classes, macroglia and Müller glial cells (ü glia), and one immune class encompassing monocytes and macrophages (myeloid) and vascular endothelial cells (VEC). This classification was based on both established canonical markers and the most significantly variable upregulated genes (Fig. 3A, B). To further understand the changes in gene expression in the retinal diabetic model, we analyzed the DEGs between the db/db and db/m groups. Our findings revealed the upregulation of the expression of genes related to inflammation (*Dusp*), ubiquitination (*Mid1* and *Trim17*), apoptosis (*Cirbp* and *Actg1*), proliferation (*Ttll11*), and autophagy (*Sgk1* and *Mir124-2hg*). In contrast, we observed the deregulation of genes encoding members of the heat shock protein family in the db/db group compared to the db/m group (Fig. 3C). The expression of genes related to apoptosis (*Cirbp* and *Anp32a*), ubiquitination (*Uba52*), inflammation (*Mif*), and glycolysis (ALDOA) were downregulated in the WIF1 group compared to the db/db group (Additional file 1: Figure A).

In addition, GO and KEGG analyses indicated that glycolysis-related pathways were significantly upregulated in cone and RBCs in the db/db group, whereas inflammation-related pathways, such as IFNG and TNF, were significantly upregulated in microglia and myeloid cells in the db/db group. Additionally, we found that visual perception and protein processing in the endoplasmic reticulum were significantly downregulated in the db/db cone-rod cell subsets and that intercellular tight junctions were significantly downregulated in the db/db vascular endothelial cell subsets (Additional file 1: Figure B, C). Glycolytic pathways that were enhanced in the db/db cone subset were significantly downregulated in the WIF1 group. Moreover, pathways such as mitochondrial oxidative phosphorylation were also downregulated in cone and RBCs, and pathways such as tight junctions and cell morphology were significantly upregulated in VEC (Additional file 1: Figure D, E). Further analysis of the upregulated and downregulated signaling pathways in the cone subsets revealed that the glycolytic, HIF-1α, and VEGF pathways were significantly activated in the db/db group but inhibited in the WIF1 group (Fig. 3D, E). Therefore, we hypothesized that the HIF-1α and glycolytic pathways may be downstream of WIF1 regulation in photoreceptor cells.

### WIF1 protects photoreceptor cells by inhibiting glycolysis in db/db mice

**Fig. 4:**
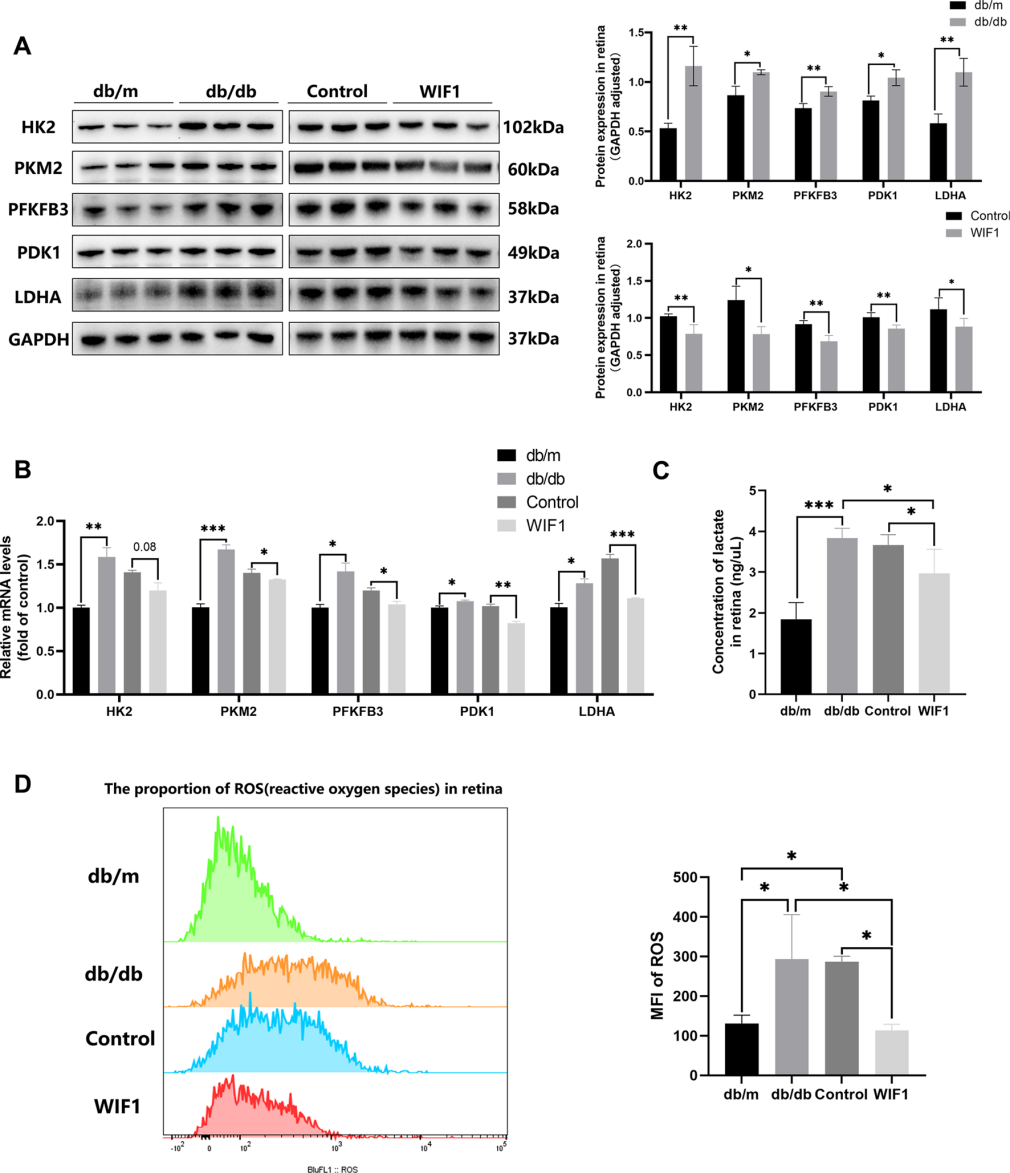
WIF 1 inhibits glycolysis and improves photoreceptor function in db/db mice. **A** Western blot and quantification of retinal protein levels in the db/m, db/db, control, and WIF1 groups. **B** qRT-PCR analysis of the *WIF1* mRNA decreased in db/db mice treated with LV-WIF1. **C** Levels of secreted lactate in db/db mouse retinas. **D** Flow cytometric analysis was employed to detect reactive oxygen species (ROS) within the retinas of the four groups of mice, followed by an assessment of mean fluorescence intensity (MFI). The presented data depict the average and standard deviation from a minimum of three repetitions, with differences assessed using standard one-way ANOVA. *p < 0.05, **p < 0.01, ***p < 0.001

To further investigate the effect of WIF1-mediated glycolysis on photoreceptor cell injury and DR, we conducted in vivo experiments using db/db mice. As shown in Fig. 4A, B, both western blotting and qRT-PCR analyses revealed an increase in the levels of glycolysis-related enzymes in db/db mice, contrasting with the effects observed upon *WIF1* overexpression. Consistent with these observations, treatment with LV-WIF1 partially decreased the expression of lactate, the end product of glycolysis [16], in the retina of db/db mice at 16 weeks of age (Fig. 4C). Hyperactivation of glycolysis can lead to the excessive generation of ROS and increased oxidative stress in the retina. Correspondingly, we measured the ROS fluorescence content in the retinas of mice and found that treatment with WIF1 reduced the oxidative stress level in the retinas of db/db mice (Fig. 4D). These results demonstrate that WIF1 can reduce oxidative stress by reducing glycolytic flux.

### WIF1 improves the function of retinal neurons in DR

**Fig. 5:**
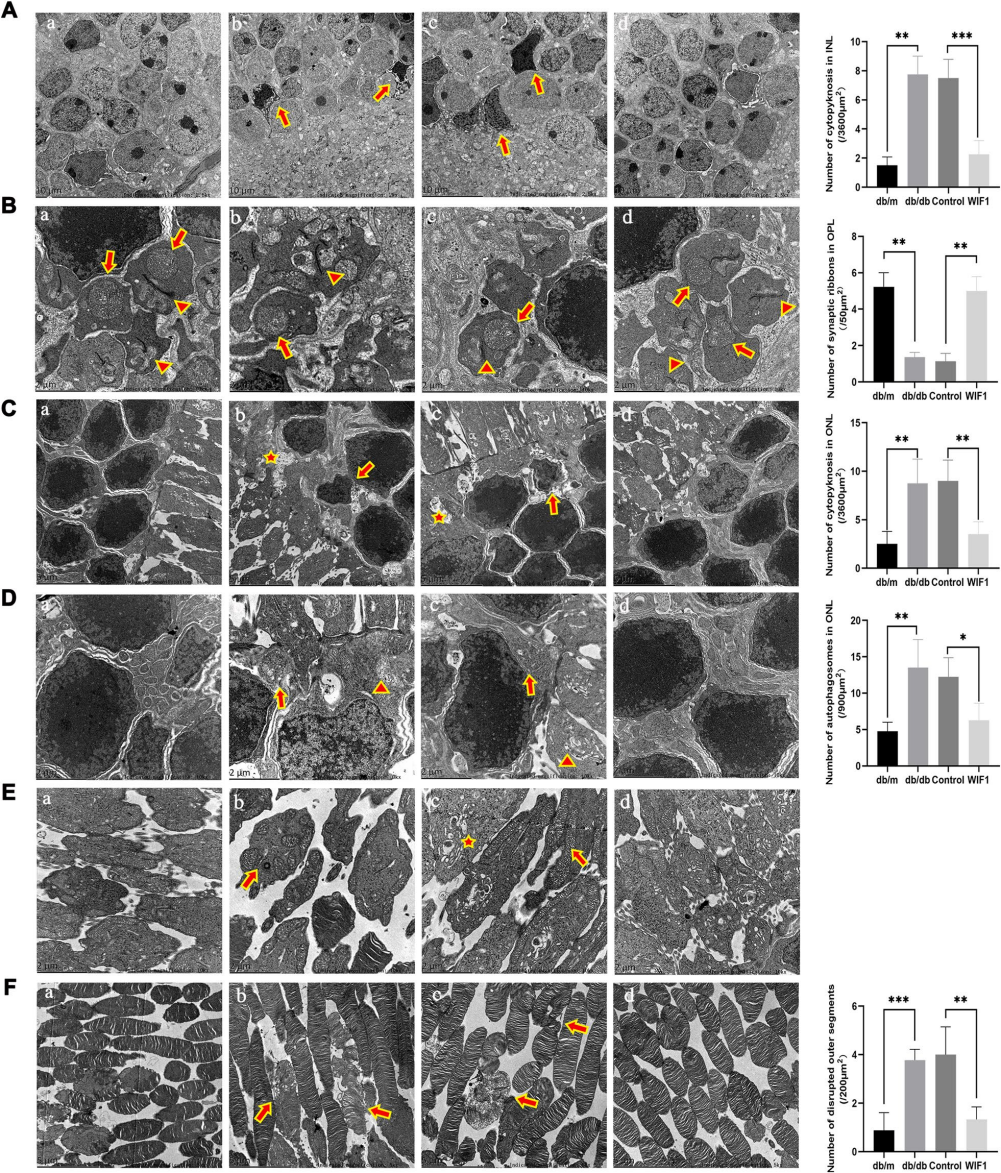
WIF1 overexpression normalizes retina histology in db/db mice. **A** TEM images depicting cytopyknosis (arrow) observed in the inner nuclear layer of db/db mice retina across various groups: (a) db/m, (b) db/db, (c) control, and (d) WIF1. **B** TEM images illustrating the presence of mitochondria (arrow) and photoreceptor synaptic ribbons (triangle) within the outer plexiform layer (OPL) of four groups: (a) db/m, (b) db/db, (c) control, and (d) WIF1. **C** TEM images showcasing autophagosomes (pentagram) and cytopyknosis (arrow) within the outer nuclear layer (ONL) of different groups: (a) db/m, (b) db/db, (c) control, and (d) WIF1. **D** TEM images displaying endoplasmic reticulum (triangle) and mitochondria (arrow) within various groups: (a) db/m, (b) db/db, (c) control, and (d) WIF1. **E** TEM images reveal Golgi structures (pentagram) and mitochondria (arrow) in photoreceptors within different groups: (a) db/m, (b) db/db, (c) control, and (d) WIF1. **F** TEM images highlighting photoreceptor outer segments (OS) (arrow) in diverse groups: (a) normal, (b) db/db, (c) control, and (d) WIF1. The presented data depict the average and standard deviation from a minimum of three repetitions, with differences assessed using standard one-way ANOVA. *p < 0.05, **p < 0.01, ***p < 0.001

We further investigated the impact of WIF1 on retinal ultrastructure using transmission electron microscopy (TEM). The inner nuclear layer of db/db mice retina exhibited cytopyknosis, with nuclei of varying sizes (Fig. 5A-b). However, when treated with WIF1, cytopyknosis in the retina of db/db mice was reduced, and an abundance of regular organelles was observed. The mitochondria and endoplasmic reticulum remained intact and clear (Fig. 5A-d). The retina of WIF1-treated db/db mice (Fig. 5B-d) contained more organelles, photoreceptor synaptic ribbons, large mitochondria in the rod spherules, and multiple mitochondria in the cone pedicles than control (Fig. 5B-c). The retinal ONL was composed of rod and cone cells with large nuclei, highly electron-dense chromatin, and visible nucleoli (Fig. 5C-a). Moreover, autophagosomes and cytopyknosis increased in the retinal ONL of db/db mice (Fig. 5C-b) but decreased in that of the WIF1 group (Fig. 5C-d). In the ONL of db/db mice, the endoplasmic reticulum showed dilation and vesiculation, mitochondrial swelling, increased mitochondrial membrane density, and reduced mitochondrial crista structures (Fig. 5D-b). In contrast, in the WIF1 group, the structure was orderly, and the endoplasmic reticulum was clear (Fig. 5D-d).

Photosensitive proteins are synthesized in the inner segment and the region adjacent to the cell body. The inner segment is enriched with rough endoplasmic reticulum, mitochondria, and the Golgi complex [17]. In db/db mouse retinas, the outer layer of photoreceptors exhibited swelling with mitochondrial hypertrophy, and the Golgi vesicles were visibly dilated and blurry compared to db/m mice (Fig. 5E-b). Conversely, the outer layer of the photoreceptors of WIF1-treated db/db presented normal mitochondria and Golgi (Fig. 5E-d). The photoreceptor cells of db/m mice demonstrated elongated and well-organized outer segment morphology (Fig. 5F-a). In contrast, db/db mice exhibited disrupted and swollen outer segments, resulting in shorter outer segment morphology (Fig. 5F-b). Overexpression of *WIF1* in the retina maintained the normal structure of the outer segment of the retinal photoreceptor cells (Fig. 5F-d).

**Fig. 6:**
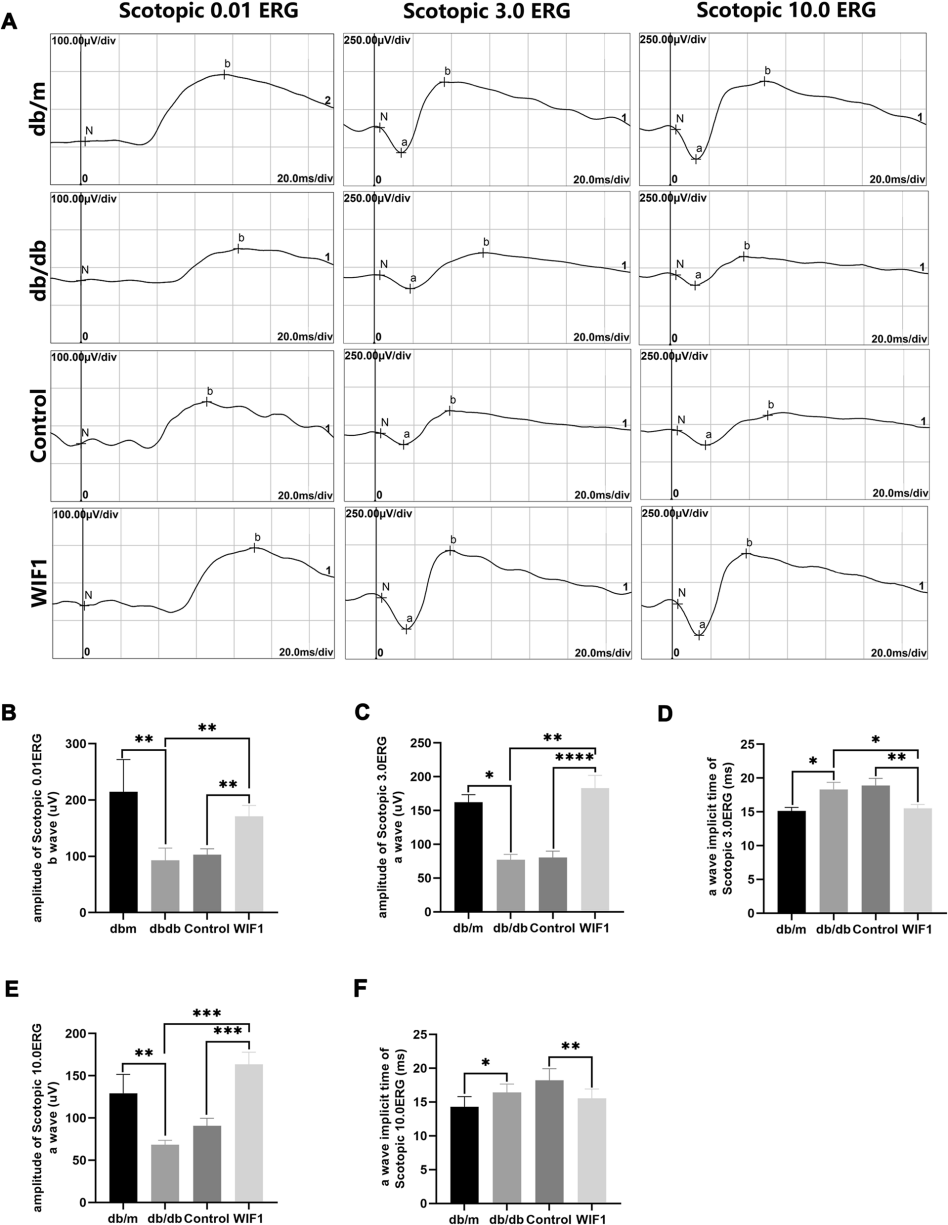
WIF1 overexpression normalizes the vision function of db/db mice. **A** Scotopic ERG amplitudes and implicit timing recorded across four groups: normal, db/db, control, and WIF1. **B** Scotopic ERG 0.01. **C** and **D** Scotopic ERG 3.0. **E** and **F** Scotopic ERG 10.0. The presented data depict the average and standard deviation from a minimum of three repetitions, with differences assessed using standard one-way ANOVA. *p < 0.05, **p < 0.01, ***p < 0.001

In addition, ERG results highlighted the impact of WIF1 on alterations in visual function. Under the guidance of the International Society for Clinical Electrophysiology of Vision (ISCEV) [18], Fig. 6A illustrates that the scotopic 0.01 ERG b-wave (indicating rod response), the scotopic 3.0 ERG test a-wave (indicating cone and rod responses), and the scotopic 10.0 ERG test a-wave (indicating cone responses) decreased, and implicit time was prolonged in db/db mice compared to db/m mice. Conversely, the overexpression of *WIF1* resulted in a significant enhancement of a-wave amplitudes, compared to those in the control group, accompanied by a reduction in the implicit time (Fig. 5B-F). Hence, WIF1 has a positive impact on protecting the ultrastructure and enhancing the function of retinal neurons in DR.

### WIF1 ameliorates HG-induced glycolytic dysfunction in 661 W cells

**Fig. 7:**
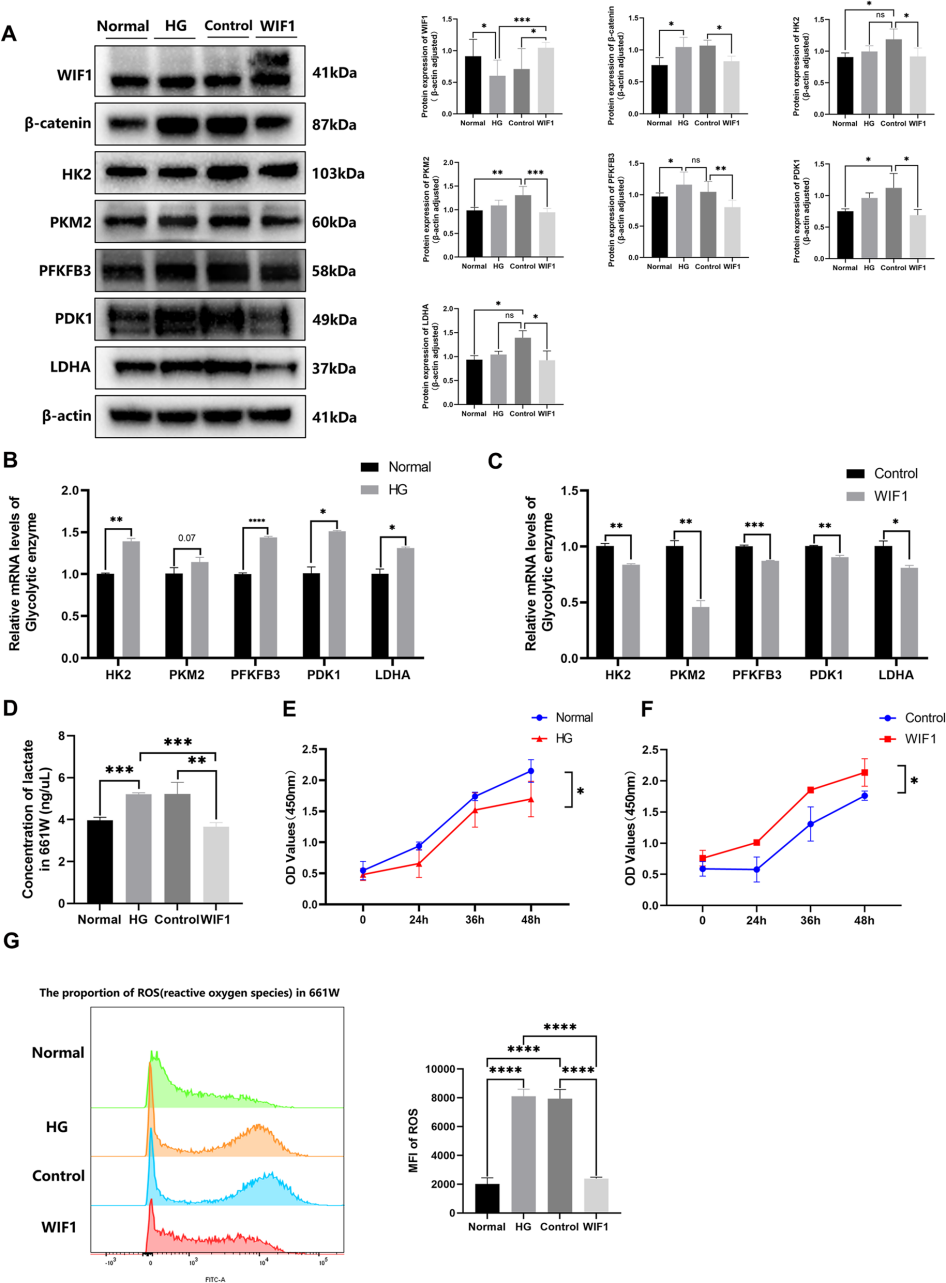
WIF1 alleviates HG-induced glycolytic dysfunction in 661 W cells and protects photoreceptor cells. **A** Western blot and quantification of HK2, PKM2 (**C**), PFKFB3, PDK1, WIF1, and LDHA protein levels in 661 W cells. **B**-**C** RT-qPCR analysis of the mRNA levels of the genes encoding glycolysis enzymes in 661 W cells. **D** Levels of secreted lactate of 661 W cells of four groups. **E**, **F** Proliferation detection via the CCK-8 assay. Results are expressed as OD values (450 nm). **G** Flow cytometric analysis was employed to detect the ROS of the four 661 W cell groups, followed by an assessment of mean fluorescence intensity (MFI). The presented data depict the average and standard deviation from a minimum of three repetitions, with differences assessed using standard one-way ANOVA. *p < 0.05, **p < 0.01, ***p < 0.001

We further assessed the expression of glycolytic enzymes and lactate secretion in vitro using the 661 W cell line, which is an important in vitro model for studying cone photoreceptor cell biology and related diseases [19]. In HG-treated 661 W cells, the levels of key glycolytic enzymes, including HK2, pyruvate kinase M2 (PKM2), PFKFB3, PDK1, and LDHA, were increased. Treatment with 3xFlag tagged LV-WIF1 inhibited the expression of β-catenin was inhibited and decreased that of glycolytic enzymes (Fig. 7A). Similarly, the mRNA levels of the genes encoding the glycolytic enzymes were decreased in the WIF1 group compared to those in the control group (Fig. 7B, C). Lactate levels were significantly increased in 661 W cells induced by HG, whereas they were significantly decreased by *WIF1* overexpression (Fig. 7D). In the cell-counting kit (CCK)-8 cell viability assay, the WIF1 group demonstrated notably enhanced growth speed compared to the control group, indicating effective control of metabolic dysfunction in the high-glucose environment (Fig. 7E, F). To elucidate the underlying mechanism of this effect, we analyzed the ROS levels and found that treatment with WIF1 significantly reduced the elevated ROS levels induced by high glucose (Fig. 7G). These results indicate that WIF1 alleviates HG-induced glycolytic dysfunction, enhances cellular viability, and reduces oxidative stress in photoreceptor cells.

### WIF1 regulates glycolysis enzymes via the HIF-1α pathway

**Fig. 8:**
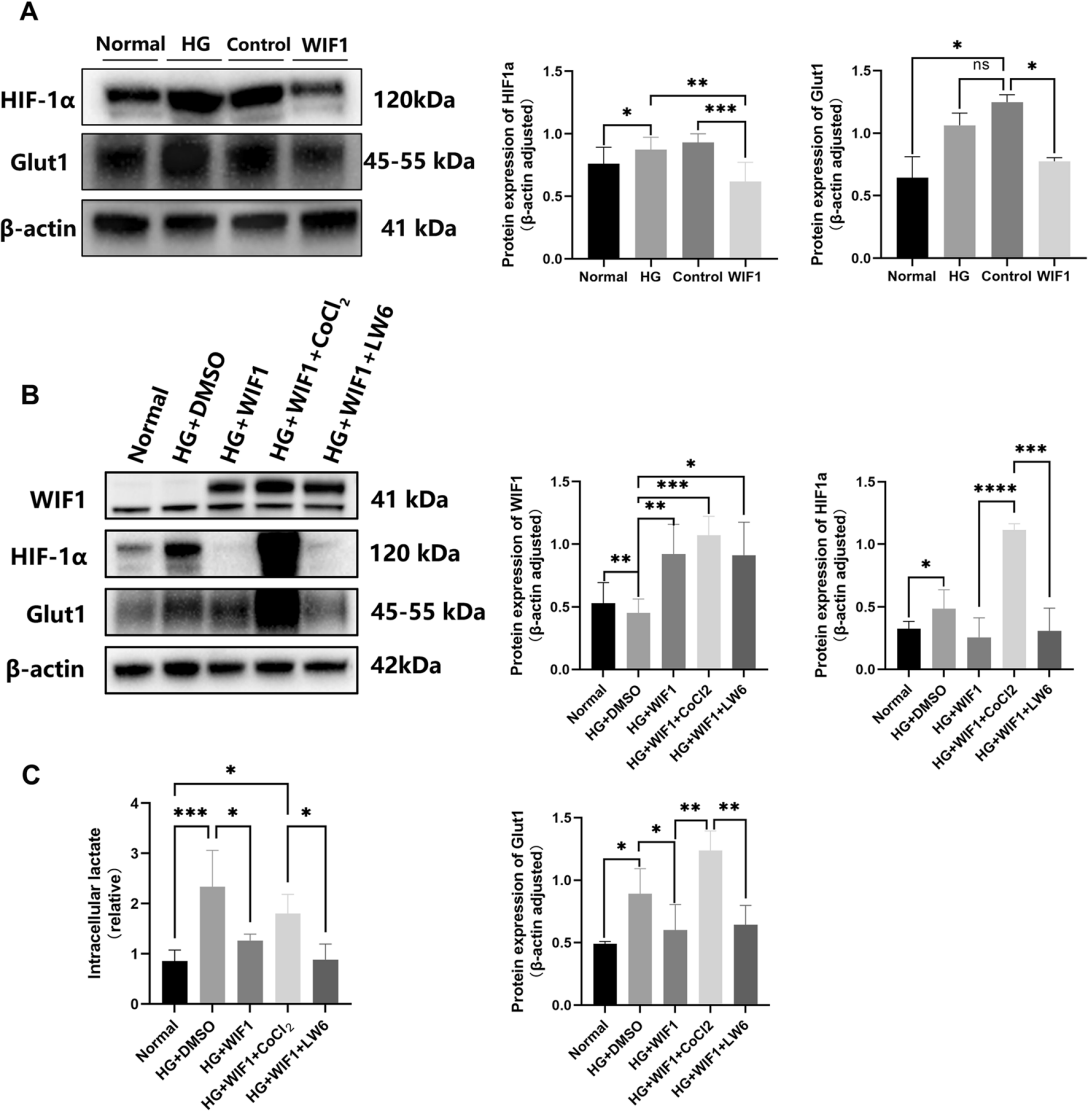
WIF1 regulates glycolysis enzymes in 661 W cells via the HIF-1α pathway. **A** Western blot and quantification of HIF-1α and Glut1 in 661 W cells. **B** Western blot and quantification of WIF1, HIF-1α, and Glut1 in 661 W cells treated with HG (50 mM), LV-WIF1(3xFlag tagged), CoC_l2_ (10 µM), and LW6 (10 µM). **C** Levels of secreted lactate of 661 W cells treated with HG (50 mM), LV-WIF1, CoC_l2_ (10 mM), and LW6 (10 mM). The presented data depict the average and standard deviation from a minimum of three repetitions, with differences assessed using standard one-way ANOVA. *p < 0.05, **p < 0.01, ***p < 0.001

To investigate the involvement of HIF-1α in the WIF1-regulated glycolytic pathway, we analyzed the expression of HIF-1α and Glut1 in 661 W cells. We observed the upregulation of HIF-1α and Glut1 expression in 661 W cells treated with high glucose, whereas their expression was suppressed in the presence of LV-WIF 1 (Fig. 8 A). We treated 661 W cells with LW6 (HIF-1α inhibitor) and CoCl_2_ (HIF-1α activator) to manipulate the levels of HIF-1α. Western blot analysis and lactate secretion assays demonstrated that WIF1 exhibited the same inhibitory effect on HIF-1α as LW6. Moreover, the expression of HIF-1α was restored by CoCl_2_ stimulation (Fig. 8B, C). Collectively, these findings suggest that WIF1 modulates the effects of glycolytic enzymes that regulate lactate production in 661 W cells by activating the HIF-1α pathway.

**Fig. 9:**
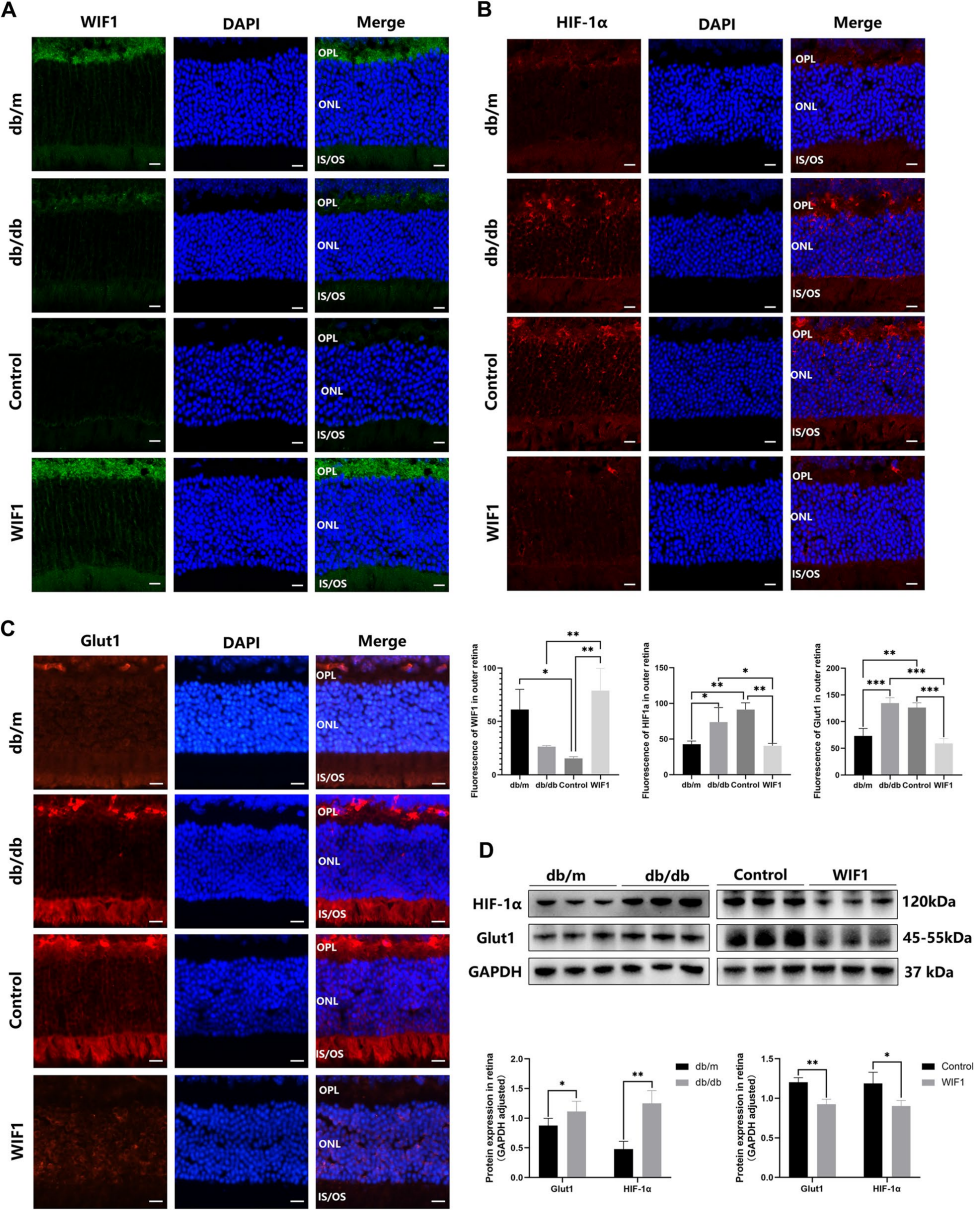
WIF1 attenuates the HIF-1α pathway in db/db mice retina. Immunostaining results depicting (**A**) WIF1, (**B)** HIF-1α, and (**C**) Glut1 expressing in the outer retinas of normal and db/db mice. The fluorescence intensities of WIF1, HIF-1α, and Glut1 staining were calculated using Image J software. **D** Western blot analysis of WIF1, HIF-1α, and Glut1 expression in db/db mice. The presented data depict the average and standard deviation from a minimum of three repetitions, with differences assessed using standard one-way ANOVA. *p < 0.05, **p < 0.01, ***p < 0.001

In addition, we performed immunofluorescence (IF) analysis to visualize and quantify the expression of WIF1, HIF-1α, and Glut1 in the retina. Notably, *WIF1* was stably and effectively expressed in LV-WIF1-treated db/db mice (Fig. 9A). HIF-1α and Glut1 were predominantly expressed in the OPL and ONL. The WIF1 group exhibited lower HIF-1α and Glut1 expression levels than the control group (Fig. 9B, C). This pathway was further validated in the db/db mice retina; western blot analysis revealed increased expression of HIF-1α and Glut1 in db/db mice, which was reversed by treatment with WIF1 (Fig. 9D).

## Discussion

The hallmarks of DM-induced neuronal degeneration include apoptosis and neuroglial degeneration. Photoreceptor cells, which are composed of cones and rods, are the predominant neuronal cell type in the retina and are essential for vision formation. Damage and loss of photoreceptor cells are the primary causes of permanent vision loss in patients with neurodegenerative eye diseases [20]. In recent years, morphological changes and thickness reductions of cones and rods have been detected in the early stages of diabetes in animal models [21, 22]. Human retinal spectral domain OCT has also shown that patients with DR have reduced photoreceptor layer thickness and that cone density significantly differs between non-DR and non-proliferative DR eyes and controls. These differences in cone density tend to increase with the duration of diabetes [23, 24]. Photoreceptor damage increases retinal oxidative stress, promotes the formation of a retinal inflammatory environment [25, 26], and leads to the destruction of the blood-retinal barrier and microvasculature [27]. Damage to photoreceptor cells may manifest during the initial phases of DR, which can also affect the regulation of diabetic retinal angiogenesis. Therefore, mitigating photoreceptor cell damage is vital for improving visual function and curbing the onset of microvascular damage.

The traditional streptozotocin-induced diabetic mouse model involves the destruction of insulin-producing cells, which results in diminished insulin secretion and the onset of diabetes. In contrast, the C57BL/KsJ-db/db mouse model spontaneously develops type 2 diabetic mellitus (T2DM) due to a mutation in the leptin receptor [28, 29]. In this model, diabetes originates from abnormalities in insulin signaling rather than from insufficient insulin secretion. Over the years, this model has become a valuable tool for investigating the underlying mechanisms of DR within the context of obesity-induced T2DM. Numerous studies have demonstrated the presence of retinal neurodegeneration, including glial activation, mitophagy, cell apoptosis, and retinal thinning in this model [30–32]. In our study, db/db mice maintained high blood glucose levels at 12 weeks of age and exhibited 2–3 times higher body weights than age-matched db/m mice, confirming the hallmark characteristics of T2DM. Therefore, the C57 BL/KsJ-db/db model is well-suited for investigating the potential mechanisms underlying DR-related retinal neurodegeneration.

WIF1, as an inhibitor of the Wnt signaling pathway, directly interacts with various Wnt ligands, hindering their binding to membrane-bound receptors [10]. WIF1 suppresses tumor vessel growth and induces cancer cell apoptosis by reducing the expression of proteins, such as c-myc, cyclinD1, stromal-derived factor-1α (SDF-1α), and VEGF [33, 34]. WIF1 is expressed during the early developmental stages of rods and in the interphotoreceptor matrix of the mature retina [11, 35]. However, its expression is downregulated in the aqueous humor of patients with proliferative DR (PDR) [13, 36]. These findings indicate that WIF1 is an attractive candidate for drug targeting in ocular diseases.

The impact of Wnt/β-catenin signaling in neurons is bidirectional, and it is important to note that the effects of Wnt ligands on axons are complex and concentration-dependent, as reported in various studies [37–39]. Different Wnt ligands have been observed to exhibit varying influences on axonal growth. Additionally, the canonical and non-canonical Wnt pathways can exhibit opposing effects, frequently inhibiting each other, thereby contributing to the intricate role of Wnt/β-catenin signaling in retinal neurons [40, 41]. Consequently, it is proposed that a potential therapeutic strategy for age-related macular degeneration and retinal degeneration involves mitigating canonical Wnt signaling by activating antagonistic non-canonical Wnt signaling pathways [42]. In DR, research on the role of the Wnt pathway has primarily focused on abnormal neovascularization and inflammatory processes [43]. Aberrant activation of Wnt signaling components has been observed in DR in several studies [44]. In DR models, the neutralization of Wnt/β-catenin signaling by kallistatin and Mab2F1 has been shown to improve ocular inflammation and mitigate retinal neurodegeneration [45, 46]. The present study demonstrates the inhibition of β-catenin four weeks after intravitreal injection of WIF1 lentivirus in db/db mice. This finding further confirms the potential of WIF1 to protect retinal neurogenesis and blood vessels by suppressing the canonical Wnt/β-catenin signaling pathway.

Furthermore, our study indicates a significantly low level of WIF1 in high-glucose-induced 661 W cells and retinas of db/db mice, highlighting the crucial role of WIF1 overexpression in maintaining photoreceptor function in the db/db mouse model. To explore the mechanism by which WIF1 protects against DR, we performed scRNA-seq and subsequently analyzed the DEGs in db/db mice. We observed the upregulation of genes related to inflammation, ubiquitination, apoptosis, proliferation, and autophagy, along with the downregulation of members of the heat shock protein family. This result suggests that these changes may lead to increased cellular apoptosis and stimulation of autophagy, playing a crucial role in the pathogenesis of DR in db/db mice. Additionally, we observed a downregulation trend in protein processing pathways related to visual perception and the endoplasmic reticulum in the cone-RBC subpopulation. This result suggests that cell types in db/db retinas may exhibit abnormalities in visual function and endoplasmic reticulum homeostasis. Endoplasmic reticulum stress and mitochondrial dysfunction have been implicated in vascular and neuronal damage in DR [47]. Metabolic dysfunction disrupts the balance between glycolysis and oxidative phosphorylation, resulting in further biochemical and molecular changes that are observed in DR. These changes include failures of communication between the endoplasmic reticulum and mitochondria and disruptions in mitochondrial autophagy. Together, these factors significantly contribute to the development of DR [48, 49]. As expected, the glucose metabolism pathway, glycolysis pathway, and the related genes were markedly upregulated in the db/db mice in the present study. The upregulation of WIF1 leads to alterations in the expression of genes related to apoptosis, ubiquitination, inflammation, and glycolysis. Particularly, in the cone cell subpopulation, the glycolysis, HIF-1α, and VEGF pathways were significantly downregulated in the WIF1 group, indicating that WIF1 plays an essential negative regulatory role in these biological processes. In conclusion, WIF1 regulates the excessive activation of the glycolytic pathway and may be a significant contributor to the alleviation of photoreceptor cell damage during the progression of DR.

Glycolysis is the main metabolic pathway in the retina, responsible for metabolizing approximately 80–96% of glucose. This process provides energy and maintains retinal cell health, particularly in photoreceptor cells [50]. Under physiological conditions, glucose in the bloodstream is transported to photoreceptor cells through the RPE via Glut1, where it is phosphorylated by HK2 to form glucose 6-phosphate (G6P). Subsequent enzymatic steps convert G6P to fructose 1,6-bisphosphate, generating ATP, pyruvate, and lactate [51]. However, under conditions of high blood glucose levels, the high levels of lactate generated by glycolysis cannot be absorbed by the RPE, leading to its accumulation in photoreceptor cells. Ignasi et al. indicated that patients with PDR exhibit elevated levels of lactate and glycolytic flux in the vitreous humor [28]. The accumulation of lactate and other metabolites can act as toxins within the cell, and the excessive production of ROS through oxidative phosphorylation can inhibit the body's antioxidant defense mechanisms and promote oxidative stress. This, in turn, can damage the nucleus, DNA, and mitochondria, thereby accelerating photoreceptor cell injury and apoptosis [48, 51]. Notably, the Wnt signaling pathway can promote intracellular glycolysis by activating HIF-1α, Glut1, HK, and PKM2 in tumor cells [29]. Glut1, a major glucose transporter in the retina, is the only known glucose transporter expressed by photoreceptors. Holman et al. reported that overexpression of *Glut1* can lead to the accumulation of glucose metabolites, resulting in mitochondrial damage and ultimately, apoptosis of photoreceptors in DR. Blocking Glut1 expression in photoreceptor cells can regulate glucose metabolism and alleviate retinal inflammation and peroxidation [52]. Similar results have been observed for downstream glycolytic enzymes [53, 54]; PFKFB3-driven glycolysis impairs the antioxidant capacity of neurons, leading to neuronal loss and reactive gliosis [8]. The present study provides evidence that the Wnt/β-catenin pathway was activated in the retina of db/db mice and HG-treated 661 W cells. This activation led to increased downstream HIF-1α expression, subsequently promoting Glut1 expression and mediating glycolysis. The accumulation of glucose metabolites results in elevated oxidative stress and a higher degree of mitochondrial damage in photoreceptor cells, which accelerates injury and apoptosis. In contrast, our results suggest that WIF1 inhibits abnormal glycolytic processes, reducing lactate production, oxidative stress, and apoptosis. Hence, we propose that WIF1 can exert a defensive function in photoreceptor cells by governing the Wnt signaling pathway, HIF-1α, Glut1, and glycolysis. This regulation helps mitigate oxidative stress and protect the integrity and function of photoreceptor cells. Consequently, WIF1 may serve as an option for early intervention in the management of DR, preserving visual function in affected patients.

Extensive population studies have consistently reported a significant decline in visual acuity and ERG responses in individuals with PDR, and the presence of neurodegeneration in all phenotypes of DR [55, 56]. The shortened and disorganized photoreceptor outer segments contribute to a reduction in a-wave amplitude [57]. In the present study, TEM imaging in db/db mice revealed that photoreceptor cells exhibited karyopyknosis-like changes in the nuclei and shorter and cluttered outer segments compared to normal mice. Furthermore, in the OPL, photoreceptor-bipolar cell synapses collect information and transmit signals to neurons in the retina's inner plexiform layer (IPL), which contains multiple ribbon synapses that serve as the beginning of visual information collection in the downstream parallel signaling pathway [58]. Synaptic reduction affects transient and persistent neurotransmitters and the release of synaptic vesicles, resulting in reduced visual information processing [59, 60]. In the present study, WIF1 restored the integrity of the outer segment structure and effectively countered the synaptic loss in the OPL. Both OCT and H&E analyses revealed that the thickness values of the retinal outer layer in db/db mice decreased over the course of DR, indicating the presence of neurodegeneration in the outer layers of the retina of db/db mice. In contrast, there was a marked increase in the thickness values of these layers in the db/m and WIF1 groups. Moreover, the observed reduction in the thickness of GCL + IPL and RNFL, comprising Müller cells and ganglion cells, suggests the presence of neurodegeneration in the inner retinal layer as well. Notably, WIF1 exhibits a therapeutic effect on neurons in the inner layer, suggesting potential avenues for future research.

Our study has some limitations. Considering that lentiviral vectors can infect all retinal cell types, further investigations using adeno-associated viruses specifically expressed in db/db mouse photoreceptor cells are warranted. Second, it should be noted that WIF1 does not fully reverse all lesions in DR. The scRNA-seq findings indicated the presence of additional cytopathic processes within the retinas of db/db mice, including the upregulation of autophagy and inflammation-related genes. Notably, microglia appear to be susceptible to the influence of inflammation-related pathways. Hence, future research is crucial to elucidate whether dysregulation of vision function in DR is also linked to microglia-mediated gliosis and neurodegeneration, as well as whether other factors are involved in the pathology of both WIF1 and DR neurodegeneration.

## Conclusions

**I**n summary, intravitreal injection of a *WIF1* lentivirus to overexpress *WIF1* in the retina modulates glycolytic enzymes through the Wnt/β-catenin-HIF-1α pathway, reducing retinal lactate concentrations in diabetic mice. WIF1 plays a key role in inhibiting glycolytic flux, accumulation of glucose metabolites, photoreceptor damage, and amelioration of retinal neuronal damage in db/db mice. This environment helps alleviate some of the pathological damage to retinal photoreceptor function and morphology compared to mice with diabetes. This study established a new theoretical basis for the treatment of DR and holds important clinical value.

### Supplementary Information

**Additional file 1: Figure S1.** Single-cell RNA sequencing reveals differentially expressed genes that were upregulated and downregulated in db/db vs. db/m and WIF1 vs. db/db in all retinas. (A) Volcano diagram showing that WIF1 downregulates the upregulated inflammatory and apoptotic genes (*H3f3b*, *Cirbp*, and *Mif*) and Wnt pathway-related genes (*Cnn-4*) in the db/db group. Genes with Log2 fold change >0.25 and *p *< 0.05 were identified as differentially expressed. (B, C) The bubble diagram shows the upregulation (red) and downregulation (blue) of the GO and KEGG signaling pathways in various retinal subsets in the db/db group compared with the db/m group (Top30). (D, E) Bubble diagram showing changes in GO and KEGG signaling pathways upregulated (red) and downregulated (blue) in various retinal subsets in the WIF1 group compared with the db/db group (Top30).

**Additional file 2: Table S1.** PCR primer list.

**Additional file 3: Table S2.** Primary antibody list.

## Data Availability

The data that support the findings of this study are available from the corresponding author upon reasonable request.

## Abbreviations

DR: Diabetic retinopathy
OCT: Optical coherence tomography
ERG: Electroretinography
TEM: Transmission electron microscopy
DM: Diabetes mellitus
WIF1: Wnt-inhibitory factor 1
ILM: Limiting membrane
RPE: Retinal pigment epithelium
TUNEL: Terminal deoxynucleotidyl transferase (TdT) dUTP nick-end labeling
CBC: Cone bipolar cell
RBC: Rod bipolar cell
AC: Amacrine cell
ü glia: Müller glia cell
VEC: Vascular endothelial cell
DEG: Differentially expressed gene
KEGG: Kyoto Encyclopedia of Genes and Genomes
IF: Immunofluorescence
