# Supplementary figures and images for: WNT-inhibitory factor 1-mediated glycolysis protects photoreceptor cells in diabetic retinopathy
Source: J Transl Med. 2024 Mar 6;22:245. doi: 10.1186/s12967-024-05046-5 (PMC10918886; doi:10.1186/s12967-024-05046-5)

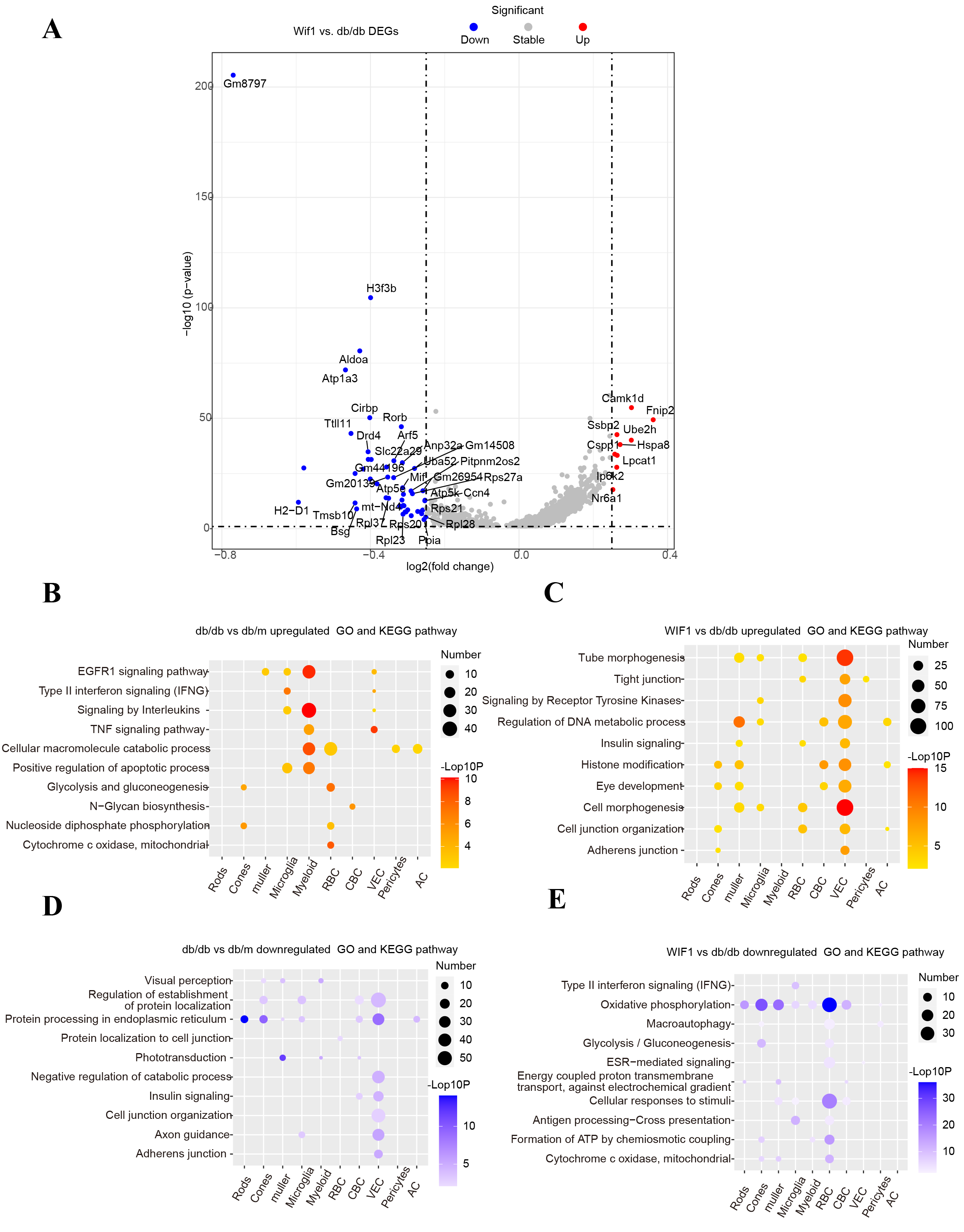

Supplement: Supplementary file 1 — Additional file 1: Figure S1. Single-cell RNA sequencing reveals differentially expressed genes that were upregulated and downregulated in db/db vs. db/m and WIF1 vs. db/db in all retinas. (A) Volcano diagram showing that WIF1 downregulates the upregulated inflammatory and apoptotic genes (H3f3b, Cirbp, and Mif) and Wnt pathway-related genes (Cnn-4) in the db/db group. Genes with Log2 fold change >0.25 and p < 0.05 were identified as differentially expressed. (B, C) The bubble diagram shows the upregulation (red) and downregulation (blue) of the GO and KEGG signaling pathways in various retinal subsets in the db/db group compared with the db/m group (Top30). (D, E) Bubble diagram showing changes in GO and KEGG signaling pathways upregulated (red) and downregulated (blue) in various retinal subsets in the WIF1 group compared with the db/db group (Top30). [file 12967_2024_5046_MOESM1_ESM.tif]
