# Supplementary material for: WNT-inhibitory factor 1-mediated glycolysis protects photoreceptor cells in diabetic retinopathy
Source: J Transl Med. 2024 Mar 6;22:245. doi: 10.1186/s12967-024-05046-5 (PMC10918886; doi:10.1186/s12967-024-05046-5)
Supplement: Supplementary file 2 — Additional file 2: Table S1. PCR primer list. [file 12967_2024_5046_MOESM2_ESM.docx]

1. **PCR primer list**

| WIF1 forward | 5’-TCTGGAGCATCCTACCTTGC-3’ |
| --- | --- |
| WIF1 reverse | 5’-ATGAGCACTCTAGCCTGATGG-3’ |
| HK2 forward | 5’-GTGTGCTCCGAGTAAGGGTG-3’ |
| HK2 reverse | 5’-CAGGCATTCGGCAATATGG-3’ |
| HIF-1α forward | 5’- ACCTTCATCGGAAACTCCAAAG-3’ |
| HIF-1α reverse | 5’- CTGTTAGGCTGGGAAAAGTTAGG-3’ |
| Glut1 forward | 5’- TCAAACATGGAACCACCGCTA-3’ |
| Glut1 reverse | 5’- AAGAGGCCGACAGAGAAGGAA-3’ |
| PFKFB3 forward | 5’- CCCAGAGCCGGGTACAGAA-3’ |
| PFKFB3 reverse | 5’- GGGGAGTTGGTCAGCTTCG-3’ |
| PDK1 forward | 5’- GGACTTCGGGTCAGTGAATGC-3’ |
| PDK1 reverse | 5’- TCCTGAGAAGATTGTCGGGGA-3’ |
| PKM2 forward | 5’- GCTCTAGGTATCGCGCAGG-3’ |
| PKM2 reverse | 5’- GTAAGCGTTGTCCAGGGTGA-3’ |
| LDHA forward | 5’-AACTTGGCGCTCTACTTGCT-3’ |
| LDHA reverse | 5’- TAGCCGCCTGAGGACTTACT-3’ |
| β-actin forward | 5’- GCAGGAGTACGATGAGTCCG-3’ |
| β-actin reverse | 5’- ACGCAGCTCAGTAACAGTCC-3’ |
