# Supplementary material for: WNT-inhibitory factor 1-mediated glycolysis protects photoreceptor cells in diabetic retinopathy
Source: J Transl Med. 2024 Mar 6;22:245. doi: 10.1186/s12967-024-05046-5 (PMC10918886; doi:10.1186/s12967-024-05046-5)
Supplement: Supplementary file 3 — Additional file 3: Table S2. Primary antibody list. [file 12967_2024_5046_MOESM3_ESM.docx]

**Primary antibody list**

| anti-WIF1 | AB186845, Abcam, USA |
| --- | --- |
| anti-β-catenin | AB32572, Abcam, USA |
| anti-GFP | AB183734 , Abcam, USA) |
| anti-HIF-1α | 36169, Cell Signaling Technology, USA |
| anti-HIF-1α | 66730, Proteintech, China |
| anti-HK2 | 2867, Cell Signaling Technology, USA |
| anti-PFKFB3 | 13763, Proteintech, China |
| anti-PDK1 | AB207450, Abcam, USA |
| anti-LDHA | 3582, Cell Signaling Technology, USA |
| anti-Glut1 | 21829, Proteintech, China |
| anti-Glut1 | 66290, Proteintech, China |
| anti-PKM2 | 4053, Cell Signaling Technology, USA |
| anti-β-actin | 20536, Proteintech, China |
| anti-GAPDH | GB11002, Servicebio, China |
